# Evaluating the Ethos automated planning system for spatially fractionated radiotherapy

**DOI:** 10.1002/acm2.70306

**Published:** 2025-10-10

**Authors:** A Aziz Sait, Yoganathan SA, Amine Khemissi, Umang Patel, Sunil Mani, Satheesh Paloor, Rabih Hammoud

**Affiliations:** ^1^ Radiation Oncology Physics Advanced Medical Physics Houston Texas USA; ^2^ National Center for Cancer Care & Research (NCCCR), Radiation Oncology Hamad Medical Corporation Doha Qatar; ^3^ Radiation Oncology Saint John Regional Hospital Saint John New Brunswick Canada; ^4^ Radiation Oncology Millennium Physicians Oncology The Woodlands Texas USA

**Keywords:** automated planning, ethos, hypoxia, IOE, Lattice radiation therapy, NSCLC, spatially fractionated radiotherapy (SFRT)

## Abstract

**Purpose:**

Lattice radiotherapy (LRT), a form of spatially fractionated radiation therapy (SFRT), has emerged as a promising approach for treating massive tumors. By delivering high‐dose regions within the tumor while sparing surrounding healthy tissue, LRT offers distinct advantages over conventional radiotherapy. Recent advancements in treatment planning systems (TPS), particularly the integration of intelligent optimization engines (IOEs) with automated planning capabilities, have the potential to further refine and expand the clinical utility of LRT. This study aimed to comparatively evaluate the planning quality and clinical feasibility of lattice SFRT treatment plans generated using the Ethos planning system, equipped with an IOE and O‐ring linear accelerator, versus the Eclipse planning system paired with a conventional C‐arm TrueBeam linac, in patients with stage III non‐small cell lung cancer (NSCLC).

**Methods:**

Twenty retrospective stage III NSCLC cases (GTV > 200 cc) with available PET‐CT imaging were selected. A total of 40 plans (20 Eclipse, 20 Ethos) were compared, incorporating lattice spheres (1 cm diameter, 2 cm spacing between spheres) placed in the tumor, FDG‐PET/CT‐informed intratumoral heterogeneity, prioritizing viable perinecrotic subregions while avoiding critical OARs. Plans aimed to deliver 15 Gy to lattice spheres, limit Valley (PTV minus spheres) doses to 2 Gy, and restrict doses to organs at risk (OARs) to ≤ 3 Gy. Dose conformity, OAR sparing, dose gradient parameters (PEDR, PVDR), planning time, and deliverability, which was evaluated using ArcCheck, EPID gamma analysis, and MLC log‐file verification.

**Results:**

Ethos demonstrated statistically significant improvements compared to Eclipse in lattice sphere mean dose (17.2 Gy vs. 15.83 Gy, *p* < 0.001), V15 Gy coverage (98.2 % vs. 91.74 %, *p* < 0.001), and dose gradient metrics (PEDR: 6.42 vs. 5.80; PVDR: 3.70 vs. 3.29; both *p* < 0.001, and VPDR: 0.131 vs. 0.135; PVDR_DVH_: 7.62 vs. 7.41). For the valley target, Ethos plans demonstrated a lower mean dose (Dmean: 4.72 Gy vs. 4.91 Gy, *p* = 0.064), although not statistically significant, and achieved significantly improved dose gradient at V7.5 Gy (14.5% vs. 16.35%, *p* = 0.019), V5Gy (30.77% vs. 34.84%, *p* = 0.006), and V2Gy (99.77% vs. 97.79%, *p* < 0.001) compared to Eclipse. Ethos achieved significantly better OAR sparing, particularly for the bronchial tree, heart, spinal cord, esophagus, and great vessels (all *p* < 0.01). Furthermore, Ethos substantially reduced planning time (36.55 vs. 95.96 min, *p* < 0.001). Both planning systems achieved high gamma passing rates (> 95%), confirming the accuracy and deliverability of the treatment plans.

**Conclusion:**

The Ethos automated treatment planning demonstrated superior lattice dose conformity, enhanced OAR sparing, and significantly faster optimization compared to Eclipse. This automated optimization capability highlights the potential of Ethos for efficient and effective lattice radiotherapy in managing massive NSCLC tumors.

## 1 INTRODUCTION

Lattice radiation therapy (LRT), originating from early grid radiation therapy, has seen a significant evolution in its approach to treating bulky tumors.^1^, ^2^ Historically, treatments have often used custom‐made grid 2D collimators,^3^, ^4^ which are primarily effective for tumors protruding from the skin. Addressing deep‐seated tumors is a complex challenge. However, with recent technological advancements, lattice radiotherapy has emerged at the forefront, leveraging sophisticated tools and powerful 3D volumetric optimization algorithms. Current intensity modulated techniques, supported by advanced planning tools such as jaw tracking collimators and multi‐leaf collimators (MLC) shaping, enable efficient creation of the desired peak‐and‐valley radiation distribution patterns, facilitating treatment even for tumors situated deep within the body.^5^

In recent years, lattice radiotherapy has garnered significant attention from the oncology community.^6^ This renewed interest coincides with advancements in systemic therapies, particularly immunotherapy.^7^ There is growing optimism that these therapies can be synergistically combined with lattice radiotherapy to leverage the abscopal and bystander effects. Such effects are believed to stimulate immune responses beyond the directly irradiated tumor zones, potentially enhancing the overall efficacy of treatment. Numerous studies have underscored this proposition, with early clinical results indicating promising outcomes and increased treatment effectiveness.^8^, ^9^, ^10^, ^11^

Non‐small cell lung cancer (NSCLC) remains challenging to manage, particularly in advanced stages where achieving effective control is difficult due to tumor spread, size, and heterogeneity. Treatment for advanced NSCLC has traditionally relied on chemotherapy and radiotherapy, with recent advancements incorporating targeted therapy and immunotherapy approaches.^12^, ^13^ Despite improvements, obtaining desired outcomes remains complex due to intrinsic and acquired resistance, especially in large tumors that make effective dosing challenging without severe toxicities.^14^ LRT represents a novel approach to treating advanced NSCLC, addressing the challenge of delivering high, effective doses to large tumors while minimizing damage to surrounding normal and critical structures. Lattice radiotherapy involves delivering radiation in a spatially fractionated pattern (SFRT), creating regions of high dose (peaks) interspersed with regions of lower dose (valleys). The peaks target specific tumor regions, whereas the valleys are spared, allowing for tissue recovery and potentially capitalizing on the biological effects of radiation, such as vascular rebuilding. Thus, LRT offers a promising adjunctive approach for advanced NSCLC, potentially enhancing local control and improving outcomes while managing the toxicities associated with large‐volume disease.^15^

In many of the latest applications, LRT is used in a two‐step process for deep‐seated bulky tumors. Initially, a single high‐dose fraction was delivered in the lattice pattern. This is followed by conventional dose prescriptions to ensure that the entire tumor receives therapeutic radiation.^16^ Additionally, LRT can be incorporated into stereotactic treatments, both in short and long fractions, which is especially valuable when critical organs are close to the target, limiting the intended therapeutic dose delivery and adding another layer of versatility to its application.^17^

Modern linear accelerators (LINAC) are increasingly equipped with sophisticated artificial intelligence (AI) capabilities in treatment planning, coupled with enhanced imaging technologies such as positron emission tomography‐computed tomography (PET‐CT), magnetic resonance imaging, and rapid cone beam computerized tomography (CBCT). Due to their adaptive nature, such advancements can be particularly beneficial for spatially fractionated radiotherapy. These state‐of‐the‐art tools ensure the precise positioning of lattice targets and facilitate swift and high‐quality treatment planning through intelligent optimization engines (IOE).^18^ Given the intricate dose heterogeneity and imperative to spare organs at risk (OAR) in lattice SFRT, it is crucial to harness these advancements.

While most existing studies and treatment planning strategies for lattice radiotherapy have relied on conventional C‐arm LINACs and traditional inverse planning techniques, the potential of IOE with auto planning capabilities with O‐ring LINAC systems remains largely unexplored. Notably, Barsing et al.^19^ reported the delivery of lattice SFRT for liver tumors using the Halcyon O‐ring LINAC, but their work was limited to manual planning within the Eclipse treatment planning system. To date, no studies have investigated the use of the Ethos IOE for lattice radiotherapy planning or assessed its feasibility for treatment delivery using the Ethos platform.

The primary objective of this study is to evaluate the performance and feasibility of the Ethos IOE for automated planning in SFRT using lattice techniques. As a secondary objective, the study aims to compare the quality and deliverability of lattice treatment plans generated by the automated Ethos IOE with those created using the traditional Eclipse/TrueBeam planning system. This investigation introduces, for the first time, the application of Ethos' automated planning capabilities specifically to lattice‐based SFRT.

## 2 METHODS

This retrospective study included 20 patients with stage III locally advanced NSCLC, selected from our institutional database between January 2021 and April 2024. All patients received SFRT. The inclusion criteria comprised patients with a gross tumor volume (GTV) exceeding 200 cc and the availability of PET‐CT imaging suitable for accurate image fusion for treatment planning.

Simulation was performed using a four‐dimensional computed tomography (4D‐CT) scan with patients immobilized in a customized vacuum‐bag (vac‐bag) body mold to ensure positional reproducibility and stability. Patients were positioned supine with arms raised above their heads, holding a T‐bar, and supported by a knee rest for enhanced comfort and immobilization. Following respiratory binning, an averaged axial CT dataset was generated and utilized for treatment planning.

### 2.1 Volume delineations

**FIGURE 1:**
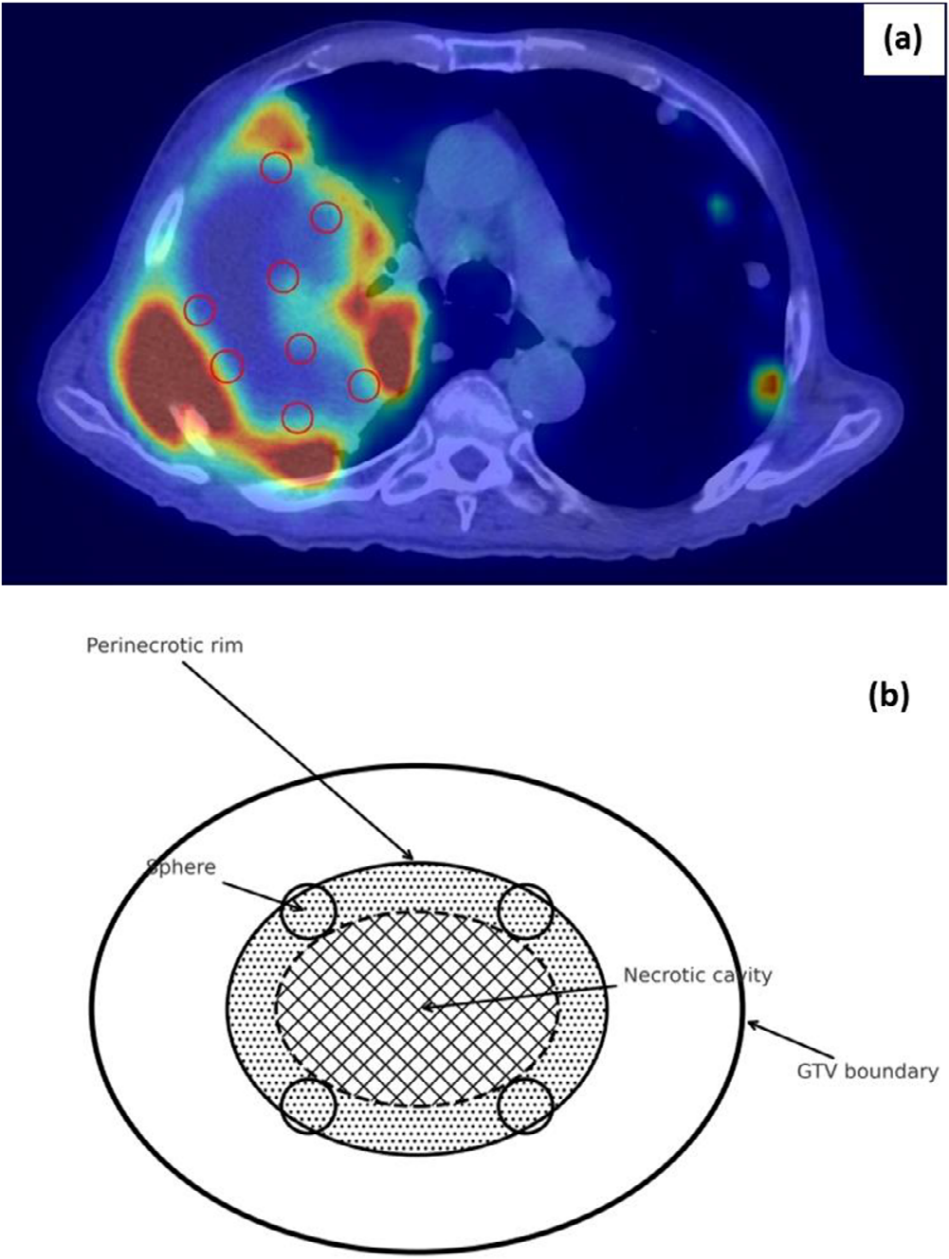
Representative ^18^F‐FDG PET/CT image of a bulky NSCLC tumor showing heterogeneous uptake. Red circles indicate lattice spheres positioned in the hypoxic prone subregion (FDG is a surrogate, not hypoxia‐validated) (a). Conceptual schematic of lattice volume design (b).

Primarily, lattice spheres were generated at least 5 mm within the GTV boundary, ensuring that no lattice spheres extended beyond the GTV and maintained a minimum separation of 1 cm from any critical OARs. Lattice spheres were placed manually using a custom design approach that considered OAR geometry and the tumor's spatial relationship to major vessels and arteries (including segmental pulmonary arteries). All patients underwent ^18^F‐FDG (Fluorodeoxyglucose) PET‐CT using our institutional standard acquisition protocol. Using fused PET‐CT imaging, lattice spheres were strategically placed within FDG/CT‐informed intratumoral heterogeneity, prioritizing viable perinecrotic subregions, corresponding to metabolically hypoxic prone areas within the PET‐defined GTV (Figure 1), each measuring 1 cm in diameter and spaced at a center‐to‐center distance of 2 cm from neighboring spheres. The total number of lattice spheres was determined based on the available volume within the GTV and the spatial relationship with surrounding OARs. Lattice spheres were contoured by expanding predefined margins around selected brush points. The spacing between spheres and their uniform placement were verified using the grid tool. The mean ± standard deviation (SD) number of lattice spheres created was 12 ± 9, corresponding to a mean lattice sphere volume of 5.9 ± 4.2 cc. The “valley” region was defined as the PTV excluding lattice spheres, with a mean ± SD volume of 847.2 ± 352 cc.

To manage and control the dose delivered to the valley regions outside the lattice spheres, an optimization target volume (OTV) was generated by PTV minus a 3 mm margin around each lattice sphere surface, facilitating gradient control. Additionally, an edge volume was contoured using an automatic wall‐extraction tool, creating a 2 mm outer ring beyond the PTV boundary, termed the “Edge.” A separate 2 cm donut‐shaped ring structure was also established at a distance of 1 cm from the PTV to further restrict dose spillage beyond the intended target area.

The OARs included in the treatment planning process were the bronchial tree, heart (inclusive of pericardium), planning organ‐at‐risk volume (PRV) of the spinal cord (3 mm margin from the spinal canal), esophagus, great vessels, ipsilateral (ipsi) lung, and contralateral (contra) lung.

In our study, an experienced radiation oncologist with extensive expertise in lattice SFRT manually contoured both the primary GTV and the lattice sphere volumes. To ensure accuracy, all contours were independently reviewed and verified by a second radiation oncologist and an experienced clinical physicist in our SFRT unit. This manual approach was chosen because it allowed for clinical judgment and multidisciplinary input, ensuring precise alignment with biologically relevant tumor subregions.

General planning objectives for treatment plan optimization with both Eclipse and Ethos systems included the following: administration of 15 Gy in a single fraction to the lattice spheres, limiting the valley dose (PTV minus lattice spheres) to 2 Gy, and also limiting 50% (7.5 Gy) of the dose to the GTV outside the lattice spheres. Maximum allowable doses for critical OARs were constrained to 3 Gy.

### 2.2 Treatment planning with Eclipse

Treatment planning was performed using the Varian TrueBeam LINAC with 6 MV Flattening Filter‐Free (FFF) mode. Eclipse photon optimizer with Acuros XB calculation (version 16.1) was utilized, with 1.25 mm calculation grid resolution and fine optimization settings. Three partial arcs were employed for treatment: 181°–29° (clockwise), 29°–181° (counter‐clockwise), and 181°–29° (clockwise).

To achieve high conformity to the lattice target, collimators were optimized using an arc geometry tool, minimizing the sharing of MLCs between tumor regions. Collimator jaws were set to ensure optimal target coverage, and jaw tracking was enabled during optimization. Each partial arc was assigned distinct collimator angles, typically set at 30° (CW), 330° (CCW), and 45° (CW); however, adjustments within 10° to 45° were made based on the geometry of the lattice targets. Using a visual MLC run‐up and arc geometry tool, collimator angles were occasionally modified to further enhance dose conformity.

For the lattice spheres, the target generalized equivalent uniform dose (gEUD) was set to 15 Gy with an “*a*” parameter of −40. A lower physical volume objective was also established to ensure that 100% of the lattice volume received 15 Gy, with no upper dose limit imposed on the lattice targets to allow dose heterogeneity. To control the Valley dose outside the lattice targets, a combination of upper gEUD (*a* = +40) and upper physical volume objectives was applied to the Valley target. Additionally, a lower physical volume objective was set to ensure that the entire PTV received a minimum of 2 Gy. For OARs, a combination of upper gEUD with an ‘*a*’ parameter of +40 and upper physical maximum dose objectives was set to maintain point doses under 3 Gy.

The automatic normal tissue objective was utilized, along with a 2 cm donut ring structure placed 1 cm away from the PTV, to control the 2 Gy dose spillover outside the PTV. Both GPU‐based and intermediate‐dose calculations were utilized during optimization. Priority weights were iteratively adjusted to achieve clinical goals while balancing doses between the target and OARs. Eighteen cases were optimized twice, while two cases underwent three optimization cycles.

During optimization,^20^ the process was closely monitored, particularly at multi‐resolution levels 1 and 2 in Eclipse TPS (version 16.1). The aim was to maintain a balance between target coverage, Valley dose gradient, and OAR dose constraints. Progress was assessed through real‐time visualizations of dose distributions and DVHs. As priority weights for Valley dose control and OAR constraints increased, any peripheral coverage shift in the target indicated progress toward the desired balance. Upon achieving this balance, the optimization transitioned to level 3 for the final dose calculation.

For both Eclipse and Ethos plans, no manual dose normalization was applied; all plans were evaluated as optimized by their respective treatment planning systems, with objectives set to achieve clinically acceptable target coverage and OAR sparing.

### 2.3 Treatment planning with Ethos

All 20 patients were imported into the Ethos planning system for treatment planning. Two target volumes were initially defined: Lattice Spheres, including all the lattice spots, and PTV, excluding the lattice spheres. The lattice spheres were prescribed a dose of 15 Gy, while the PTV was prescribed 2 Gy. To deliver the prescribed 15 Gy dose to the lattice spheres, certain parts of the PTV would receive higher doses, potentially exceeding 150%–250% of the prescribed dose. This creates high‐dose hotspots within the PTV, which the current version (v2.1) of the Ethos system cannot handle, as it does not support hotspots exceeding 150%. This limitation can interfere with the automatic plan generation process.

**FIGURE 2:**
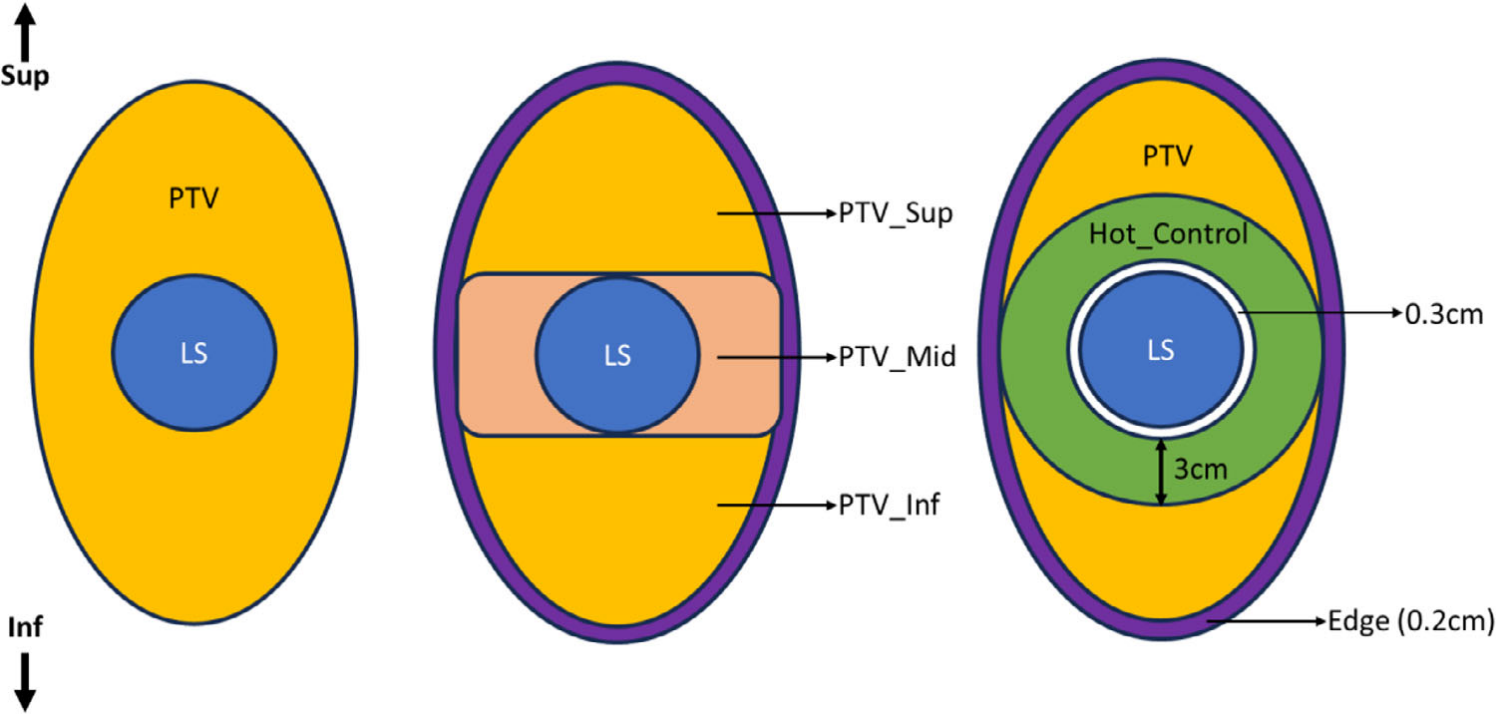
Visual representation of the volume partition used for Ethos planning.

To address this issue, a new approach was developed. The PTV was divided into two regions: PTV_Mid and PTV_Inf+Sup. The PTV_Mid was defined as the portion of the PTV within the axial plane of the lattice spheres and was expected to receive a higher dose due to the GTV. A dose of 5 Gy was prescribed to this region. The remaining portion of the PTV (excluding PTV_Mid) was labeled PTV_Inf+Sup, and this region was prescribed a dose of 2 Gy. Additionally, two rings were created around the lattice spheres to help control the high‐dose hotspots. The first ring, called “Hot_control,” was positioned 3 mm from the Lattice Spheres with a 3 cm wall thickness, and the second ring, called “Edge,” was a smaller wall placed 2 mm from the PTV. These adjustments were implemented to better manage the hotspot in the high‐dose area. A visual representation of the volume partition is provided in Figure 2.

The Hot_control region was intentionally kept localized rather than elongated. This was done to focus specifically on the area where we expect the most significant high‐dose fall‐off from the lattice spheres. The goal was to capture and monitor the high‐dose gradient zone immediately surrounding the lattice boost areas without extending into regions of the PTV that were not expected to receive elevated doses.

After defining the contouring task, the treatment planning process proceeded. The Ethos TPS uses an IOE for automated planning. This engine simplifies the optimization process by defining clinical goals for both the targets and the OARs, typically using predefined templates. Table 1 outlines the clinical goals used in this study. The IOE generates objective functions for the photon optimizer, allowing planners to adjust the priorities of clinical goals during optimization. During this process, real‐time visualizations of the dose distribution and dose‐volume histograms are provided for a 9‐field IMRT plan. Once the planner is satisfied with the plan, it is approved, and the final auto‐plan is generated.

**TABLE 1:** Clinical goals and planning objectives used for Ethos and Eclipse optimization.

Ethos	Eclipse
**GTV**: D99% > 100%–99% **PTV_Mid**: D20cc < 5.25–5.50 Gy D97% > 1.95–1.90 Gy **PTV_Inf+Sup**: D97% > 1.95–1.90 Gy D0.1cc < 2.10–2.20 Gy	**GTV**: Target gEUD (D15 Gy), *a* = −40; Lower (D100% of 15 Gy)
**All OARs**: Dmax < 2–3 Gy **Body‐PTV**: V2Gy < 3%–3.5 % **Edge**: Dmean < 2–2.2 Gy	**All OARs**: Upper gEUD (D3 Gy), *a* = +40; Upper (D3 Gy) **Ring**: Upper gEUD (D2 Gy), *a* = +40; Upper (D2 Gy)
**Valley (PTV‐GTV)**: Dmean < 2.5–3 Gy V7.5 Gy < 5%–8 % V5Gy < 20%–21 % **Hot_control**: D0.1cc < 12–13 Gy	**Valley (PTV‐GTV)**: Upper gEUD (D5 Gy), *a* = +40; Lower (D100% of 2 Gy) Automatic NTO

Ethos offers several auto‐plan options, including three fixed‐field IMRT configurations (7, 9, and 12 equally spaced fields) and two VMAT plans (2 and 3 full arcs). Custom beam geometries from other TPS systems, such as Eclipse, can also be imported into Ethos. For this study, beam geometries created in Eclipse, those having 3–4 partial arcs depending on the complexity of the cases, were imported into Ethos for final plan generation. The final dose calculation was performed using the Acuros XB calculation algorithm with a grid size of 2.5 mm.

In Ethos, the isocenter was assigned automatically by the TPS, typically at the geometric center of the combined target volumes, whereas in Eclipse, the isocenter was manually placed by the planner to optimize beam arrangement and minimize field openings. Although the isocenter locations were not identical, all plans were generated using the same objectives, ensuring clinically comparable dose distributions independent of isocenter placement.

gEUD‐based optimization objectives were applied in Eclipse to enhance regional dose control; this feature is not available in the current version of the Ethos TPS, and therefore was not used in Ethos planning.

### 2.4 Evaluation

The comparison parameters for the lattice targets included the mean dose (Dmean in Gy), V15Gy (%), representing the volume receiving 15 Gy in percentage, and V13.5 Gy (%), indicating the volume receiving 13.5 Gy in percentage. For the Valley region within the GTV, parameters assessed included Dmean (Gy), V7.5 Gy (%), V5Gy (%), and V2Gy (%). For the Edge structure, the Dmean (Gy) was evaluated. Dose spillage beyond the PTV was assessed using the V2 Gy (%) parameter for the body minus PTV volume. The maximum dose (Dmax in Gy) for critical structures such as the bronchial tree, heart, PRV spinal cord, esophagus, and great vessels was also evaluated. Additionally, mean dose (Dmean in Gy) for the ipsilateral (ipsi) and contralateral (contra) lungs, monitor units, and treatment planning time (in minutes) were recorded. The planning optimization time was calculated from the moment of plan template insertion to the point at which the optimization goals were balanced and finalized in the calculation. Time for target and OAR delineations was not included in the reported planning time.

The peak‐to‐edge dose ratio (PEDR) was defined as the ratio of the mean dose to the lattice target over the mean dose to the 2‐mm Edge structure, while the peak‐to‐valley dose ratio (PVDR) was calculated as the ratio of the mean dose to the lattice target over the mean dose to the Valley region.^21^, ^22^ A higher PVDR indicates stronger spatial dose modulation, meaning higher relative dose escalation in lattice spheres while keeping valley doses low; conversely, lower PVDR suggests weaker modulation. PEDR quantifies the sharpness of the dose fall‐off at the boundary of the target. A higher PEDR represents a steeper gradient (better confinement of hot spots within the target), while a lower PEDR indicates broader dose spill into surrounding tissue. Thus, PEDR complements PVDR by assessing gradient quality at the target margins.

Additionally, some studies, such as Grams MP, et al.^23^ reported a DVH‐based PVDR defined as D10/D90, where D10 represents the near‐maximum dose to lattice spheres and D90 represents the near‐minimum dose in the valley region. This definition avoids reliance on point doses (Dmax, Dmin) and better captures the heterogeneity across the dose volume histogram. A higher D10/D90 PVDR indicates stronger peak–valley heterogeneity.

Furthermore, the Radiosurgery Society (RSS) GRID/Lattice working group Zhang H, et al.^24^ recommended reporting the reciprocal, valley to peak dose ratio VPDR = D90(valley)/D10(peak), to ensure consistency and avoid single‐point overestimation. In this convention, a lower VPDR reflects deeper valleys relative to peaks (better modulation), while higher VPDR values indicate reduced modulation.

Statistical significance was determined using paired *t*‐tests, with a significance level of *p* < 0.05, performed using Jamovi statistical software version 2.3.

The deliverability of the treatment plans was verified on the respective treatment machines using patient‐specific QA devices. The Ethos plans were delivered on the machine, and the ArcCheck device was utilized to measure the delivery accuracy. Additionally, the MLC log files were recorded and processed using Mobius3D for further analysis. The delivered fluence was compared with the TPS fluence using a gamma evaluation with criteria of 3% (global) and 2 mm to assess the agreement between the planned and delivered doses. Eclipse‐optimized plans were delivered on the TrueBeam linac, where verification was performed using the onboard electronic portal imaging device (EPID‐DMI) and MLC log files verification with an in‐house MATLAB program.

## 3 RESULTS

**FIGURE 3:**
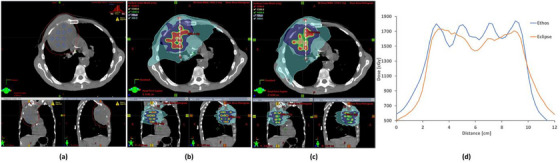
Comparison between Ethos and Eclipse. (a) Contours showing lattice spheres (blue) and valley regions (red). Corresponding dose distributions for Ethos (b) and Eclipse (c). (d) Vertical dose profile extracted along the white dotted line indicated in (b) and (c).

**FIGURE 4:**
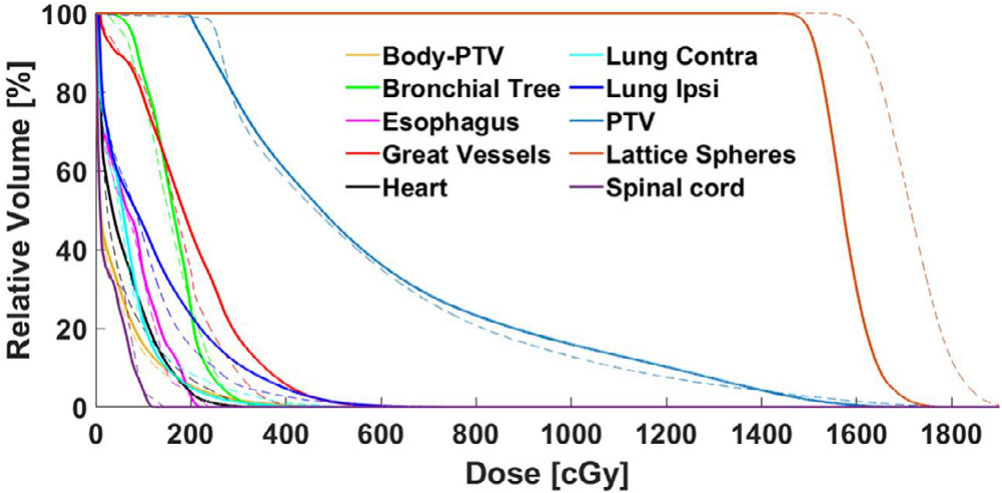
DVH Comparison between Ethos (dashed line) and Eclipse (solid line) for the targets and critical organs. (no plan normalization method).

Tables 2 and 3 present a comparison of the DVH results for the Ethos and Eclipse treatment plans. As shown in Tables 2 and 3, Ethos demonstrated an advantage over Eclipse, particularly in sparing critical OARs and reducing planning time. Coverage of the lattice sphere targets was slightly better with Ethos, along with improved Valley dose gradient control compared to Eclipse planning. Figure 3 illustrates the lattice dose distribution, highlighting the Peak and Valley profiles, while Figure 4 presents the DVH comparison for targets and critical OARs.

**TABLE 2:** DVH analyses of the target.

DVH Parameters	Ethos (Mean ± SD)	Eclipse (Mean ± SD)	*p*‐value
**Lattice Spheres**			
Dmean (Gy)	17.2 ± 0.50	15.83 ± 0.69	< 0.001
V15Gy (%)	98.2 ± 2.94	91.74 ± 5.56	< 0.001
V13.5 Gy (%)	100 ± 0	99.99 ± 0.04	0.33
**Valley target**			
Dmean (Gy)	4.72 ± 0.65	4.91 ± 0.74	0.064
V7.5 Gy (%)	14.5 ± 5.78	16.35 ± 6.83	0.019
V5Gy (%)	30.77 ± 9.14	34.84 ± 11.33	0.006
V2Gy (%)	99.77 ± 0.53	97.79 ± 1.40	< 0.001
**Edge**			
Dmean (Gy)	2.70 ± 0.23	2.76 ± 0.31	0.159
**Outside PTV spillage**			
V2Gy (%)	5.08 ± 1.27	5.43 ± 1.74	0.105
**MU**	4962.7 ± 684	4329.9 ± 625	< 0.001
**Planning time (minutes)**	36.55 ± 7.67	95.96 ± 14.89	< 0.001
**PEDR**	6.42 ± 0.58	5.80 ± 0.66	< 0.001
**PVDR (mean)**	3.70 ± 0.48	3.29 ± 0.48	< 0.001
**VPDR(D90/D10)**	0.131 ± 0.00430	0.135 ± 0.00709	0.005
**PVDR*_DVH_* (D10/D90)**	7.62 ± 0.250	7.41 ± 0.393	0.006

**TABLE 3:** DVH analyses of the OARs.

DVH Parameters	Ethos (Mean ± SD)	Eclipse (Mean ± SD)	*p*‐value
Bronchial tree (Dmax Gy)	3.54 ± 0.92	3.99 ± 0.82	< 0.001
Heart (Dmax Gy)	3.54 ± 1.54	3.86 ± 1.62	< 0.001
PRV spinal cord (Dmax Gy)	2.16 ± 0.59	2.69 ± 0.69	< 0.001
Esophagus (Dmax Gy)	2.75 ± 0.77	3.13 ± 0.76	< 0.001
Great vessels (Dmax Gy)	3.79 ± 1.46	4.15 ± 1.27	< 0.001
Ipsi‐lung (Dmean Gy)	1.18 ± 0.78	1.22 ± 0.78	0.309
Contra‐lung (Dmean Gy)	0.41 ± 0.19	0.41 ± 0.17	0.541

The comparison between Ethos and Eclipse plans for the lattice target revealed the following mean differences: Ethos produced a higher lattice Dmean by 1.38 Gy (*p* < 0.001) and an increased lattice V15 Gy by 6.46% (*p* < 0.001). Additionally, Ethos plans required 633 more monitor units (*p* < 0.001) but reduced planning time by 59.41 min (*p* < 0.001) compared to Eclipse. The PEDR and mean‐based PVDR values were also higher with Ethos, showing differences of 0.621 (*p* < 0.001) and 0.415 (*p* < 0.001), respectively. In contrast, the VPDR (D90/D10) and PVDR*_DVH_* (D10/D90)_,_ showed smaller mean differences of 0.004 (*p* = 0.005) and ‐0.205 (*p* = 0.006), respectively. These results suggest that while Ethos demonstrated higher peak‐to‐valley and PEDRs, indicating stronger peak enhancement, the DVH‐based metrics reflected slightly improved overall dose heterogeneity compared to Eclipse.

For the valley target, Ethos plans demonstrated a lower mean dose (Dmean: 4.72 Gy vs. 4.91 Gy, *p* = 0.064), although not statistically significant, and achieved significantly improved dose gradient at V7.5 Gy (14.5% vs. 16.35%, *p* = 0.019), V5Gy (30.77% vs. 34.84%, *p* = 0.006), and V2Gy (99.77% vs. 97.79%, *p* < 0.001) compared to Eclipse. The edge‐region mean doses (Dmean) were similar between both systems (2.70 Gy Ethos vs. 2.76 Gy Eclipse, *p* = 0.159), with no significant difference observed. Additionally, outside‐PTV spillage (V2Gy) was slightly lower for Ethos, but not statistically significant (5.08% Ethos vs. 5.43% Eclipse, *p* = 0.105), indicating comparable performance regarding dose spillage control.

When it came to sparing OARs, Ethos plans outperformed Eclipse across several key structures. Notably, Ethos achieved meaningful dose reductions to OARs, lowering exposure to the bronchial tree by 0.36 Gy (*p* < 0.007), the heart by 0.26 Gy (*p* < 0.009), and the PRV spinal cord by a substantial 0.53 Gy (*p* < 0.001). Similar trends were observed for the esophagus (0.37 Gy reduction, *p* < 0.001) and great vessels (0.36 Gy reduction, *p* < 0.001), highlighting Ethos's consistent advantage in minimizing the dose. Although dose reductions to the ipsilateral (0.035 Gy, *p* = 0.309) and contralateral lungs (0.01 Gy, *p* = 0.541) were modest and not statistically significant, the overall pattern points to Ethos as the more OAR‐conscious planning approach.

The 3D gamma passing rate showed that both Ethos and Eclipse‐optimized plans achieved gamma passing rates above 95%. Log file QA verification also yielded gamma passing rates above 95%.

All plans and volumes were visually inspected, and DVHs were evaluated by both physicists and radiation oncologists to ensure accuracy and clinical feasibility.

## 4 DISCUSSION

Our study demonstrated that LRT for advanced NSCLC can be effectively optimized using the Ethos IOE and delivered through the O‐ring linac. Recent studies have demonstrated that the Ethos automated planning system provides comparable or improved planning outcomes relative to conventional Eclipse and semi‐automated RapidPlan optimization methods for complex radiotherapy techniques, including stereotactic treatments.^25^, ^26^, ^27^, ^28^ However, the capabilities of Ethos for SFRT, particularly lattice‐based approaches, have not been extensively investigated. Our comparison of Eclipse and Ethos planning optimizations revealed that Ethos provided an advantage in target coverage and achieved better OAR sparing, with statistically significant improvements for most OARs. Importantly, the automated optimization capabilities in Ethos led to significantly reduced planning times compared to Eclipse, where traditional inverse planning requires a more manual approach to reach a balanced plan. This reduction in planning time highlights the efficiency gains possible through the integration of AI‐driven planning systems.^29^

The evolution of LRT has benefited greatly from the introduction of automation, particularly in the generation of lattice patterns through advanced scripts and algorithms that streamline the formation of lattice segments.^30^, ^31^, ^32^ Ethos, with its automated planning workflows, not only enhances the speed of planning but also improves consistency in lattice segment positioning, contributing to a more standardized approach to LRT. Recent developments have focused on adaptive radiation therapy, where Ethos offers distinct advantages with its adaptive capabilities and advanced imaging integrations, Hypersight CBCT, which allow for real‐time modifications to the treatment plan in response to anatomical changes.^33^

The adaptability provided by Ethos could be particularly advantageous for fractionated lattice stereotactic treatments in various bulky cancerous tumors. The ability to reproduce precise lattice geometries across treatment fractions ensures that therapeutic effects are consistently delivered to the defined targets while sparing surrounding normal tissue, maintaining the balance of high‐dose peaks and low‐dose valleys throughout treatment. This is especially relevant in advanced lung cancer, where anatomical changes due to tumor shrinkage or physiological shifts can impact the accuracy of conventional non‐adaptive treatments.^34^ Ethos's adaptive planning not only compensates for these changes but also allows for enhanced imaging‐based tracking, enabling high precision in lattice delivery for complex cases.

In the management of large, painful metastatic tumors, minimizing on‐table time is essential for patient comfort. The Ethos platform provides a practical advantage through its adaptive workflow, which reuses pre‐defined clinical goals and optimization priorities during each session. This reuse allows rapid on‐table adaptation and significantly reduces the time required for subsequent adaptive plans. In addition, Ethos offers faster CBCT acquisition and verification compared with conventional C‐arm linacs, further streamlining the workflow. We believe this makes Ethos a feasible and efficient solution for adaptive SFRT treatments, particularly when delivering a high‐dose single fraction followed by conventionally fractionated therapy. In our clinical experience, substantial tumor shrinkage is often observed after approximately 15 fractions, and the ability to adapt plans in real time using onboard CBCT can minimize the need for repeat simulations and re‐planning.

In our study, arc geometry differences between the two systems can be noted. Because the primary objective was to evaluate the performance of Ethos automated treatment planning, a fundamentally different platform from Eclipse, we allowed flexibility in planning parameters to reflect each system's standard clinical workflow and strengths. Our intent was not to directly compare identical inputs, but rather to assess how each platform performs under realistic, clinically representative conditions. Since the optimization strategies differ significantly between Eclipse and Ethos, varying beam geometries were sometimes required for each platform to achieve clinically acceptable plans.

Regarding the calculation grid size (CGS), we agree that CGS can influence dose calculation accuracy, especially for small fields or SBRT. Ethos uses a default grid size of 2.5 mm (reducible to 2 mm), which facilitates fast optimization and is particularly important for online adaptive workflows. In contrast, Eclipse allows finer grid resolution (1.25 mm), which we initially used in our study. To address this concern, we recalculated five Eclipse plans using a 2.5 mm CGS to match Ethos and compared the dosimetric outcomes. The results showed minimal differences, with the mean change across all analyzed DVH parameters not exceeding 0.5%, even for high‐gradient regions. Given the large lattice PTV volumes and the distribution of many 1‐cm spheres, such small discrepancies in CGS had a negligible clinical impact.

In this study, no dedicated hypoxia‐specific PET tracers (e.g., ^18^F‐FMISO (Fluoromisonidazole), ^18^F‐FAZA (Fluoroazomycin arabinoside), ^18^F‐HX4 (Flortanidazole)) were used in this cohort. We acknowledge that hypoxia‐specific tracers would provide a more direct and standardized method for identifying hypoxic subregions,^35^, ^36^, ^37^ and we plan to incorporate such imaging in future clinical cases to further evaluate its impact on SFRT planning and treatment response.

For the present work, ^18^F‐FDG PET‐CT was used as a surrogate map of intratumoral heterogeneity; thus, our method should be regarded as PET‐informed rather than hypoxia‐validated. Because FDG does not specifically bind hypoxic cells and low uptake may also indicate necrosis, sphere centers were deliberately placed within the viable perinecrotic rim adjacent to low‐attenuation, low‐FDG cavities, while avoiding overt necrosis.

This approach aligns with the classic Thomlinson–Gray model of diffusion‐limited hypoxia, in which a necrotic core is surrounded by a rim of viable hypoxic cells that exhibit relative radioresistance.^38^ By targeting this rim, we aimed to escalate the dose to regions most likely to benefit from peak‐dose intensification. Thomlinson and Gray also noted that delivering a sufficient dose to the more radiosensitive outer tumor layers could improve oxygenation of previously hypoxic regions, enhancing radiosensitivity for subsequent fractions. This reoxygenation phenomenon supports the combined strategy of delivering a high‐dose lattice fraction followed by conventionally fractionated radiotherapy.

Large NSCLC tumors with severe central necrosis and hypoperfusion are often associated with poor prognosis,^39^, ^40^ and are suitable candidates for lattice SFRT, which can selectively intensify dose while limiting exposure to surrounding normal tissues. As tumor growth outpaces angiogenesis, cells at the greatest distance from functional vasculature experience chronic hypoxia, contributing to radioresistance and treatment failure.

We applied conventional QUANTEC dose constraints (EQD2‐based) as the benchmark,^41^ while following ALARA principles, particularly since many patients receive systemic agents (immunotherapy/chemotherapy) in combination with radiotherapy.

Further research is needed to explore the feasibility of planning and delivering lattice Stereotactic Body Radiation Therapy (SBRT) using online, real‐time AI‐driven adaptive methods. Such studies would assess the capability to reproduce lattice target segmentations and adapt treatment plans in real time, addressing anatomical changes and ensuring precise, consistent lattice dose distributions across treatment fractions. This approach could enhance the accuracy and effectiveness of lattice SBRT by leveraging adaptive techniques to dynamically adjust the treatment plan to the patient's current anatomy, potentially improving outcomes in complex, bulky tumors.

Our initial comparison study demonstrated the Ethos system's optimization capabilities for single‐fraction LRT in NSCLC lung tumors. Most advanced lung cancer cases reported thus far have utilized single‐fraction, high‐dose LRT followed by conventional dose patterns. This approach has promising potential for application in various other anatomical sites, which could be explored in future studies.

## 5 CONCLUSION

This study demonstrated that the Ethos platform, with its advanced optimization capabilities, effectively performed treatment planning for single‐fraction lattice radiotherapy in advanced NSCLC. Compared to Eclipse optimization, Ethos achieved superior sparing of critical organs‐at‐risk, improvements in target coverage, and significantly reduced planning time due to its automated optimization process.

## AUTHOR CONTRIBUTIONS

*Conceptualization*: A Aziz Sait. *Methodology design*: A Aziz Sait. *Data acquisition*: A Aziz Sait, Yoganathan SA, and Amine Khemissi. *Treatment planning*: A Aziz Sait, Yoganathan SA, and Amine Khemissi. *Quality assurances*: A Aziz Sait, Amine Khemissi, and Sunil Mani. *Dosimetric analysis*: A Aziz Sait and Yoganathan SA. *Software implementation*: A Aziz Sait and Yoganathan SA. *Manuscript drafting and critical revisions*: A Aziz Sait, Yoganathan SA, and Umang Patel. *Led the development and execution of the study*: A Aziz Sait. *Critical revisions guidance on adaptive radiotherapy techniques*: Yoganathan SA. *Critical review of treatment planning strategies*: Yoganathan SA. *Provided clinical expertise in radiation oncology*: Umang Patel. *Patient selection criteria*: Umang Patel. *Clinical relevance validation of lattice SFRT applications*: Umang Patel. *Assisted in clinical interpretation*: Umang Patel. *DVH analysis*: Umang Patel. *Supervision of physics implementation*: Sunil Mani. *Critical review of analysis methods*: Sunil Mani. *Contributed to the interpretation of statistical analysis and outcome assessments*: Sunil Mani. *Additional data collection*: Satheesh Paloor. *Involvement in the data analysis and proofreading of the manuscript*: Satheesh Paloor. *Overall guidance, provided technical and clinical support for the treatment planning in Ethos, and a critical review of results. Contributed to the assessment of the gamma index and plan deliverability validation*: Rabih Hammoud

All authors have read/ reviewed and approved the final manuscript and agree to be accountable for all aspects of the work.

## CONFLICT OF INTEREST STATEMENT

No conflicts of interest to disclose.

## Data Availability

The data that support the findings of this study are available from the corresponding author upon reasonable request.
